# Sixth Africa malaria day in 2006: how far have we come after the Abuja Declaration?

**DOI:** 10.1186/1475-2875-5-102

**Published:** 2006-11-07

**Authors:** Joas B Rugemalila, Charles L Wanga, Wen L Kilama

**Affiliations:** 1 Multilateral Initiative on Malaria (MIM), P O Box 33207, Dar es Salaam, Tanzania; 2 African Malaria Network Trust (AMANET), P O Box 33207, Dar es Salaam, Tanzania

## Abstract

Each year on the 25th April Africa and the rest of the world commemorate Africa Malaria Day as was agreed upon at the African Summit on Roll Back Malaria held in Abuja, Nigeria on 25th April 2000. The summit also called upon the United Nations to declare the period 2001–2010 a decade for malaria. The 1^st ^Africa Malaria Day was commemorated with the theme "Communities Play a Central Role in Tackling Malaria". The 6^th ^Africa Malaria Day was observed in 2006 with the theme "Get Your ACT Together" and the slogan "Universal Access to Effective Malaria Treatment is a Human Right". This article by the Secretariat of the Multilateral Initiative on Malaria (MIM) was also part of the commemorations for the day. MIM was founded in 1997 as an alliance of institutions and individuals concerned with the malaria problem, and aiming at maximizing the impact of scientific research on malaria through strengthening African research capacity and coordinated global collaboration. The MIM Secretariat has been hosted in rotation by the founding institutions, and is being hosted for the first time in Africa by the African Malaria Network Trust (AMANET) in Dar es Salaam, Tanzania. This article reviews the malaria situation in Africa six years after the Abuja Declaration, highlighting the disease burden trends, failures, achievements, challenges, and the way forward.

## Background

Malaria is a preventable disease that afflicts hundreds of millions of people causing among them untoward socio-economic suffering including a vicious circle of abject poverty, brain damage, other irreversible disabilities, and over one million deaths per year. Notwithstanding this leading disease burden, malaria has yet to get the status it deserves on the political and other relevant agenda of endemic communities and development partners. The historic Abuja Declaration on Malaria promulgated by the Summit on Malaria in Abuja, Nigeria on 25th April 2000, like many other preceding ones, remains mostly on the drawing board as demonstrated by available statistics [1].

As Africa and the rest of the World commemorate the Africa Malaria Day on 25 April 2006 it should be noted among other observations that there is yet much to be done in sub-Sahara Africa where malaria exalts the greatest toll. No more than 60% of those suffering from malaria have prompt access to and are able to use correct, affordable and appropriate treatment within 24 hours of onset of symptoms [2]. Only 60% of the most vulnerable populations of pregnant women and children under-five years of age is estimated to benefit from the most effective combination of personal and community protective measures such as insecticide treated nets and other interventions. For example, it is estimated that of all pregnant women, only 60% has access to chemoprophylaxis by presumptive intermittent treatment as Africa Malaria Day is being commemorated for the sixth time since 2001. This article explores the current situation and what is being done to address the malaria problem.

## Malaria as a public health crisis

In Africa the annual economic burden of malaria has been estimated to be about US $12 billion, and to slow economic growth by about 1.3%. Besides contributing to loss of life, malaria morbidity may cause anaemia and its various complications, miscarriage, brain damage, decreased cognition, and productivity. It hampers children's education and social development through sickness absenteeism and neurological disabilities caused by severe infections. Adults debilitated by the disease either cannot work or do so at decreased capacity, and therefore lose earnings. Furthermore, the education system becomes disrupted when children are either too sick to attend school, or their teachers are absent because of malaria-related illnesses [3,4].

The malaria toll is staggering for every 40 seconds a child dies of malaria, resulting in a daily loss of more than 2,000 young lives worldwide. It is the leading cause of mortality and morbidity worldwide being estimated to cause 300 to 500 million clinical cases every year and between one and three million deaths, mostly among children [4]. Ninety per cent of those who die of malaria are in Africa, where the disease accounts for about one in five of all childhood deaths. Children who survive severe malaria may develop brain damage and become cognitively disabled. Malaria infections may also interact with other afflictions such as HIV infection, and under-nutrition, in ways that are still not well understood.

## Malaria historical perspectives

Some circumstantial evidences based on people movements suggest that man and malaria have evolved together, and that most, of today's populations of human malaria parasite species may have had their origin in West Africa. Hippocrates was the first to describe malaria manifestations and related them to the time of year and to where the patients lived. Before this time the manifestations were blamed on supernatural powers. The association with stagnant waters now known to be breeding sites for Anopheles mosquito vectors, led the Romans to begin drainage programmes, which was therefore the first intervention against malaria. The name malaria, meaning bad air, has its origins there.

The first recorded malaria treatment dates back to 1600, when the bitter bark of the cinchona tree used by the native Peruvian Indians to treat fevers, came to the attention of Europe through the Jesuits. The cinchona tree bark extract has since provided quinine, which is now the treatment of choice for severe malaria. Similarly, 2000 years ago the Chinese developed a fever medicine from the wormwood *Artemisia annua *from which artemisinins are nowadays extracted for the Artemisinin Combination Therapy (ACTs) being adopted as first line treatment for malaria. The plant has also become a new cash crop in some parts of East Africa.

The aetiological agent of malaria was in 1889 elicited to be a protozoa by Laveran while working in Algeria, and the *Anopheles *mosquito was demonstrated in 1897 to be the vector for the protozoa. At this point the major features of the epidemiology of malaria seemed clear, and control measures started to be implemented. In East Africa for example, during the early 1900s European colonizers took quinine regularly as preventive treatment. At the same time they started controlling mosquito breeding in towns where they mainly lived, mines, and farms supplying goods to European industries [5].

The Second World War (WW II) was a boon to the malaria control efforts by bringing the warring powers into the tropics where malaria was rife. It was, therefore, essential to discover, develop and deploy new malaria control tools in order to protect the forces exposed to malaria and other tropical diseases. So it was part of the war effort that led to the introduction of such new malaria control products as Dichloro-diphenyl-trichloroethane (DDT) and related insecticidal chemicals. Chloroquine and its relative drugs, which later constituted the mainstay of malaria prevention and control were also part of the war efforts to provide malaria interventions. Even sulphadoxine-pyrimethamine (SP) was developed in order to contain chloroquine resistant malaria during the Vietnam War. After the WWII both DDT and chloroquine entered civilian use. Since these interventions were so efficacious and effective they were adopted for use in community wide malaria control programmes. By the 1950s there was so much confidence in the prowess of these tools, that an all out war to eradicate malaria from the entire world was declared mainly relying on DDT and chloroquine.

## What went wrong?

Despite initial malaria eradication success in Europe and North America, elsewhere success was mixed. In Latin America and many parts of Asia, there were control successes rather than eradication of the disease, as were in only a few African areas in the southern fringes, high altitude areas, and islands far from the continent. However, in many parts of Africa particularly in the savannas, malaria control success was really very limited. As a result, and taking into account the historical tumultuous changes of the early 1960s, malaria eradication in Africa was totally abandoned. The countries in Asia and Latin America were implementation of the interventions was sustained, show very obvious differences between their malaria picture versus that in Africa.

Recently African countries that have reverted to DDT use have seen spectacular successes in their malaria control efforts. These include South Africa, Mozambique, Zambia, Madagascar and Swaziland who within two years of starting DDT programmes, slashed their malaria rates by 75 percent or more. With fewer people getting sick, access to ACT drugs should be more feasible to nearly all victims, which should also cut malaria rates even further. Other African countries should learn from these shining examples, and start using DDT instead of sitting on the fence appeasing environmentalists who appear to care less about the lives of others.

## Why the failure?

Malaria is a classic of the neglected diseases, characterized by a high disease burden in the developing world, a low disease burden in high-income nations, and a low level of funding in relation to the disease burden. As with other neglected diseases, the perceived lack of a lucrative consumer market for antimalarial products explains the relatively low rate of research and development (R&D) investment by the private sector. This situation has therefore necessitated government support to be the cornerstone of malaria R&D funding [6].

As stated earlier, many of the best antimalarial medicines benefited from war research efforts and investments. Not infrequently such investments ceased during years of peace. The development of insecticidal chemicals enjoyed great investments not only during the war years, but also during peace. Indeed much of this investment was initially meant for agricultural purposes. Quite often chemicals that were successfully used in agriculture were later tried for public health use before introducing the successful ones for community wide disease control.

The pyrethroids used today on the insecticide treated nets (ITNs) illustrate the point well. They were introduced in agriculture during the 1970s. In the following decade (1980s) trials of ITNs started, and by the mid 1980s scientists working in Gambia and Tanzania published papers demonstrating their efficacy in mosquito control. By the early 1990s there were already publications confirming their protection of human beings against malaria. Unfortunately, it is only now, close to three decades after the introduction of pyrethroids in agriculture, that attempts are being made to scale up the use of ITNs for real public health impact.

Furthermore, the impact of these insecticides cannot be expected to last for ever; indeed there is already good scientific evidence predicting their future failure. When they fail, if the global insecticidal arsenal has not changed, there will be no fall back position. As the chemical industry is least interested in developing protective products for the poor, and governments, particularly of malaria endemic countries which need the alternative are more or less incapacitated, there might be real grave danger when pyrethroid resistance increases to the point of affecting control programmes. The only hope on the horizon is the Bill and Melinda Gates Foundation, which recently provided a grant to the Liverpool School of Tropical Medicine to develop a completely new class of safe insecticides to provide a fall back position.

## Malaria resurgence

Continental sub-Saharan Africa was never a part of the global malaria eradication programme. The severity of the disease, density and efficiency of *Anopheles gambiae *mosquito vectors, feasibility of eradicating the disease over such a large land mass with recurrent reinvasions, high costs, and subsequent maintenance, must have all contributed to the lack of will to undertake a continental eradication programme. Also, the eradication programme period coincided with the colonial and immediate postcolonial periods, during which little or no indigenous capacity was available to initiate and sustain malaria eradication. After a period of laissez faire regarding malaria control, these countries have had to face the re-emergence of the disease.

In recent years, reported malaria cases have been rising especially in sub-Saharan Africa. This rise could be attributed to the improved coverage of Health Information Systems (HIS), and misdiagnosis due to the general rise in fevers associated with other diseases like HIV/AIDS. However, for countries with more robust data the rise in malaria cases remains strong suggesting that the scale of the malaria problem is escalating. Some of the often mentioned reasons for this resurgence include among others; deteriorating health sectors within the region, a breakdown in malaria control efforts, rising drug and insecticide resistance, population movements, and environmental changes favouring increased malaria transmission [7]. The rises in drug and insecticide resistance deserve special mention. It is known today that malaria resistance to drugs started in East Africa during the late 1970s. Eventually chloroquine was completely lost in the late 1990s, so the change from chloroquine to other drugs happened during the early years of this millennium. This delayed decision surely caused many unnecessary deaths. Already there is a mounting wave of SP resistance [8]. Surely Africa and the rest of the world community cannot afford more disastrous delays in policy changes. Admittedly, there are many important questions begging for answers including whether there is enough knowledge about the disease and its determinants, enough tools, adequate resources, and whether governments and populations of disease-endemic countries are adequately prepared? Much as important as these questions are, they do not warrant a neglect on investing sufficiently in the fight against malaria as Africa's leading public health enemy.

## Success stories

Amid the malaria deaths and sufferings, progress is being made. African countries in 2000 committed themselves to providing by the end of 2005 prompt and effective treatment and insecticide-treated nets (ITNs) for 60% of the people at highest risk of malaria and intermittent preventive treatment (IPT) for 60% of pregnant women. Initially, implementation of these measures was severely limited by a shortage of resources for procurement of commodities. But the situation in some countries is improving. Some countries have reached or exceeded at least some of these targets with recent increases in funding from new sources. Most of the remaining countries are now poised to begin scaling up, although substantial challenges remain.

With respect to prompt and effective treatment, surveys have shown that on average half of African children with fever are treated with an antimalarial drug. Unfortunately, most of these treatments still involve chloroquine to which the parasite *Plasmodium falciparum *show very high resistance rates. Increasing both availability and accessibility to ACTs constituting the new and highly effective treatment against falciparum malaria, are expected to improve treatment outcomes within the next few years. By the end of 2004 about 23 African countries had changed their national drug policy and adopted ACTs.

With respect to progress on prevention, the number of ITNs distributed during the past 3 years has increased 10-fold in more than 14 African countries. Subsidized or free-of-charge ITN distribution has proved successful in increasing coverage of the most vulnerable populations. In most African countries, many more households have mosquito nets not treated with insecticide than ITNs. Scaling up of insecticide re-treatment services will, therefore, also be an important factor in increasing ITN coverage. The recent introduction and manufacture of permanently treated nets is expected to greatly improve overall efficacy and effectiveness.

Efforts to prevent the silent but significant burden of asymptomatic infections in pregnant women residing in areas of stable malaria transmission have been revitalized through partnerships between malaria and reproductive health programmes. A total of 11 African countries, in addition to scaling up delivery of ITNs to pregnant women, are now in the process of implementing intermittent preventive treatment (IPT) for pregnant women [9].

## New techniques against an old scourge

Over the last three decades there has been considerable interest in research and development of malaria vaccines. Research results that have been obtained so far show that malaria vaccine candidates would differ not only in their biological properties, but also in their eventual applications. Pre-erythrocytic stage vaccines also called sporozoite vaccines would generally prevent malaria infections. Asexual stage malaria vaccine candidates also called blood stage vaccines would prevent development of the disease. Sexual stage (gametocytes) malaria vaccine candidates also referred to as transmission blocking vaccines would block malaria transmission.

There is clearly a need to take advantage of ongoing advances in scientific research especially in biotechnology and related endeavours to develop the badly needed malaria vaccines. In order to sustain such efforts and ensure their eventual deployment in malarious communities it is absolutely essential that African researchers participate fully in the creation of the new products so as to ensure their progress in the entire product development pipeline. However, examination of the malaria vaccine development process reveals that all malaria vaccine discoveries, patenting, pre-clinical testing, are undertaken in well endowed northern institutions. Similarly, the current Good Manufacturing Practice (c-GMP) manufacture of test products is restricted to the north. Even early phase malaria vaccine testing is carried out in the north. Only products that are safe in very preliminary testing are tested in African populations.

## Capacity building

Achieving victory over malaria in Africa requires a new internationally funded effort dedicated to training and supporting a critical mass of African malaria researchers for their parallel involvement with researchers in the North for successful implementation of new research findings for reversing the situation in malaria endemic countries. Although there are several dozen malaria research institutions in Africa only a handful of those with strong historical links to northern institutions are ever involved in the testing of malaria vaccine candidates. Institutions lacking such links, most of which are African owned by ministries of science and technology, or ministries of health, or universities may be better placed to translate new knowledge from research into effective intervention tools. However, these are often short of essential personnel, equipment, infrastructure, and are therefore avoided in product development. The need to strengthen these neglected sites is obvious.

## Malaria R & D

For too long the global community has been reluctant to invest sufficient resources in fighting malaria, leaving it near the bottom of the world's health agenda. However, with the recent gradual increase in international awareness of the problem, malaria is now on the agenda of the health community, political arena, and international financial institutions.

In 1997, the Multilateral Initiative on Malaria (MIM), an alliance of agencies, institutions, and governments, was formed to maximize the impact of scientific research on malaria through capacity building in Africa and global collaboration. The following year, the World Health Organization (WHO), United Nations Children's Fund (UNICEF), United Nations Development Program (UNDP), and the World Bank, launched the Roll Back Malaria (RBM) Global Partnership to coordinate efforts in fighting malaria. RBM today involves 90 countries, companies, and other organizations. It recently published its "World Malaria Report 2005" on progress toward its goal of halving the burden of malaria by 2010.

## Multilateral Initiative on Malaria (MIM)

MIM is a global alliance of organizations and individuals, funding partners, and four autonomous constituents comprising the MIM Secretariat, MIM and the Special Programme for Research and Training in Tropical Diseases (MIM/TDR, MIM Communication Network (MIMCom), and Malaria Research and Reference Reagent Resource Center (MR4). The MIM mission is to maximize the impact of scientific research on malaria in Africa, through research capacity building and global collaboration, and coordination [10]. The MIM alliance is playing a critical role in the emergence of a growing body of reference research institutions staffed by well-trained national scientists and working well-rounded teams and partnerships. Most of the scientists supported by MIM ever since its inception are now holding key posts in academia, health ministries, and international organizations, where they are helping shape national and international malaria agendas and also facilitating improved and effective malaria control in Africa.

MIM is also contributing to sustainable research capacity in Africa in other various ways by providing through MIMCom mechanisms for communication and information sharing, MIM/TDR opportunities for collaborative/multicenter research, MR4 access to well-defined, standardized reagents, MIM Secretariat dissemination of information regarding research opportunities and findings through the MIM Pan-African Malaria Conferences.

International donors recently pledged $3.7 billion to The Global Fund for AIDS, Tuberculosis and Malaria (GFATM) for 2006 and 2007. The amount represents about half of the $7 billion it says it will need to fund all of its programmes for the two-year period. GFATM provides nearly three-quarters of all the money spent on malaria control including procurement of drugs and ITNs,, and has committed about $1 billion toward that end over the next two years. In 2004, it switched its support from general antimalarials to the purchase of ACTs by governments receiving its grants. Over the next two years, GFATM is expected to provide about 145 million ACT treatments [11]. Since GFATM began disbursing funds in 2003, the demand for combination therapies based on artemisinin has increased rapidly and led to a drug shortage in late 2004. In order to ensure the quality of the drugs, WHO and the United Nations Children's Fund (UNICEF) have established a mechanism to prequalify manufacturers, and are calling on countries to use only WHO-approved ACTs.

The G8 got behind an £800 m fund to battle malaria; the Bill and Melinda Gates Foundation announced still more money to hunt down a vaccine against this disease. Many other philanthropic organization and institutions have emerged to lend hands in the battle against malaria. Such august institutions include GlaxoSmithKline (GSK), Wellcome Trust, Fogarty International Center (FIC) of the of the US National Institutes of Health (NIH), London School of Hygiene and Tropical Medicine (LSHTM), African Malaria Network Trust (AMANET), Multilateral Initiative on Malaria (MIM), Malaria Vaccine Initiative (MVI), European Malaria Vaccine Initiative (EMVI) and the list continues.

## What is the way forward?

Malaria is an important social, economic, and developmental problem affecting individuals, families, communities, and countries. The best chance for successfully combating the disease requires collaboration not only of those responsible for control and research but also many other sectors, including for example agriculture, industry and commerce, transport, judiciary, education, youth, gender, children, and of course finance and planning. The research crucially important south-south, south-north, and north-north collaborations, are being promoted by MIM.

Among the ideas being nourished regarding research training is the initiative that would focus on competitively awarded long-term grants that would be dedicated to developing new "centres of research excellence" in malaria endemic areas of Africa. These centres would serve as hubs for training new scientists and assembling interdisciplinary teams for conducting major malaria research. In addition, an *African malaria research and control forum *will be established to translate malaria research results into action, discussions on malaria control success stories, research priorities, and advocacy for increased goodwill and investments in malaria research and control.

The MIM Secretariat has been ably hosted by the Wellcome Trust (1997–1999), Fogarty International Centre (FIC) of the US NIH (1999–2002) and lately by Stockholm University and Karolinska Institutet (2003–2005). The African Malaria Network Trust (AMANET) whose mission is to promote capacity strengthening and networking of malaria R&D in Africa is hosting the MIM Secretariat from January 2006 to December 2010.

## Authors' contributions

JBR conceived the article, participated in its design, coordination, and helped to draft the manuscript. CLW participated in the article design, coordination, carried out literature search, and helped to draft the manuscript. WLK participated in the design of the article and, helped draft the manuscript. All authors read and approved the final manuscript.
